# Oncolytic Virus-Based Immunotherapies for Hepatocellular Carcinoma

**DOI:** 10.1155/2017/5198798

**Published:** 2017-04-20

**Authors:** So Young Yoo, Narayanasamy Badrinath, Hyun Young Woo, Jeong Heo

**Affiliations:** ^1^BIO-IT Foundry Technology Institute, Pusan National University, Busan 46241, Republic of Korea; ^2^Research Institute for Convergence of Biomedical Science and Technology, Pusan National University Yangsan Hospital, Yangsan 50612, Republic of Korea; ^3^Department of Internal Medicine, College of Medicine, Pusan National University and Medical Research Institute, Yangsan 50612, Republic of Korea; ^4^Biomedical Research Institute, Pusan National University Hospital, Busan 49241, Republic of Korea

## Abstract

Hepatocellular carcinoma is highly refractory cancer which is resistant to conventional chemotherapy and radiotherapy, carrying a dismal prognosis. Although many anticancer drugs have been developed for treating HCC, sorafenib is the only effective treatment, but it only prolongs survival duration for about 3 months. Recently, oncolytic virotherapy has shown promising results in treating HCCs and the effects can be more enhanced by adopting immune modulatory molecules. This review discusses the current status of treating HCC and the effective strategy of oncolytic virus-based immunotherapy for the treatment of HCCs.

## 1. Introduction

Hepatocellular carcinoma (HCC) is the third most common cancer with a leading cause of cancer-related death worldwide and is the only carcinoma with increasing mortality [1, 2]. The major pathophysiological characteristics of HCC are chronic liver disease with cirrhosis. Etiology of HCC ranges from hepatitis B virus (HBV) and hepatitis C virus (HCV) infections to metabolic diseases such as nonalcoholic fatty liver disease (NAFLD) and nonalcoholic steatohepatitis (NASH) [3]. In the last 20 years, the incidence of HCC has increased 62% and over 750,000 new cases are annually identified [4, 5]. Current treatments for HCC have a lot of limitations, because survival is not guaranteed even for patients with localized HCC. For the earliest stage, tumor curative treatments such as resection and percutaneous ablation are feasible, and a 5-year survival rate in these patients ranges from 40% to 70% [6]. However, tumor recurrence for 3 years is observed in 70% of patients after resection or radiofrequency ablation (RFA). Liver transplantation is an effective treatment for cirrhosis and early tumors, but most patients are ineligible because organs are scarce [7]. Moreover, approximately more than 70% of patients with HCC are not eligible for these procedures because most have intermediate or advanced stage disease at the time of diagnosis. For intermediate stage tumors, transarterial chemoembolization (TACE) (conventional or drug-eluting beads) is the standard of care [8], but overall survival is usually less than 20 months [9, 10]. For patients with advanced HCC, the survival is dismal because median overall survival is about 7 months [11]. With the multikinase inhibitor sorafenib, the only approved systemic therapy for HCC, this survival can be increased by about 3 months [6]. Besides sorafenib, other target agents, such as sunitinib, brivanib, or linifanib, have not been proven to be superior to sorafenib [12, 13]. Several newer molecules have been shown to confer a survival advantage in a subset of patients [14]. The mean survival of patients with advanced stage HCC is less than 1 year. Most of the target agents are not tumoricidal but tumor-static agents and do not have long lasting antitumor effects after discontinuation. The major factor contributing to such a grim outcome in HCC treatment is the lack of effective therapeutics so far.

Therefore, HCC is considered a highly refractory cancer and is resistant to conventional chemotherapy. To overcome these issues, the focus is shifting from antiangiogenic therapy [15] to novel modalities to improve survival for this deadly disease [16, 17]. HCC is an attractive target for immunotherapy, because of its immunological characteristics like chronic inflammation, with several immunologic mechanisms such as evasion of immune response, immunosuppressive environment, and T cell exhaustion, which are at play to promote HCC development and growth. Several novel approaches geared towards manipulating the immune response to HCC have suggested a therapeutic benefit in early stage clinical trials [18]. Contemporary clinical studies are ongoing to evaluate the efficacy of immunotherapy to reduce the risk of relapse after surgical or percutaneous ablation and prolong survival in intermediated and advanced HCC as monotherapy or in combination.

This review discusses the current status and the barriers of immunotherapy against HCC and effective immunotherapy strategies using oncolytic virus for HCC.

## 2. Chemotherapy

Treatment options for HCC are divided into three categories as follows: (1) surgical therapies (i.e., resection, cryoablation, and liver transplantation), (2) liver-directed nonsurgical therapies (i.e., percutaneous ethanol injection (PEI), radio frequency ablation (RFA), TACE, radiation, and radioembolization), or (3) systemic nonsurgical therapies (chemotherapy, molecular-targeted therapy, and hormone therapy). Since surgical therapies and liver-directed surgical therapies are generally used in the early stages of HCCs and HCC is typically diagnosed late in the course of patient with chronic liver diseases [19], here we will discuss systemic treatment approaches for patients with advanced HCCs. It is reported that the efficacy of cytotoxic chemotherapy is generally limited [20]. Although few randomized trials have been conducted, median survival in all of the studied population has been short (less than 12 months in all cases). Main reasons for this are because of the following: (1) high rate of expression of drug resistance genes such as p-glycoprotein, glutathione-S-transferase, heat shock proteins, and mutations of p53 [21, 22]. (2) Survival is often determined by the degree of hepatic dysfunction, and systemic chemotherapy is usually not well tolerated by patients with significant underlying hepatic dysfunction. (3) Clinical investigations have been undertaken in diverse patient populations. For example, Asian patients are usually younger with well-compensated cirrhosis due to chronic hepatitis B and/or C, while North American or European patients with HCC are typically over 60 years old with alcoholic cirrhosis and comorbid illness [23]. (4) Chemotherapy may be less effective overall in patients with significant cirrhosis [24].

### 2.1. Doxorubicin and Mitoxantrone

Doxorubicin is the most studied chemotherapy agent for advanced HCC. Among the agents tried, doxorubicin-based regimens appear to have the greatest efficacy with response rates of 20–30% and a minimal impact on survival [25]. Mitoxantrone has a similar antitumor efficacy as doxorubicin (response rate 10 to 25%) [26, 27]. Antitumor effect of doxorubicin may be potentiated by tamoxifen [28]. Although 12 out of 38 patients with HCC received doxorubicin plus tamoxifen (32%) that achieved a response, the median progression-free survival was only seven months [29].

### 2.2. Fluoropyrimidines

5-Fluorouracil (5-FU) has broad antitumor efficacy with acceptably low toxicity. Although the response rate with only 5-FU has been low, combination with leucovorin can bring response rates to as high as 28% [30].

### 2.3. Gemcitabine, Irinotecan, and Thalidomide

Gemcitabine has modest activity at best. It is reported that 5 of 28 patients that received gemcitabine had a partial response of short duration (~13 weeks) [31]. Irinotecan treatment resulted in one partial response for seven months among 14 patients with advanced HCC [32]. A second trial in 29 patients showed no objective partial responses, but 12 patients had disease stabilizations [33]. Thalidomide, an agent with antiangiogenic activity, showed low rate of objective antitumor activity, with disease stabilization in up to one-third of the patients [34].

### 2.4. Cisplatin-Based Combination Therapy

Cisplatin-based combination therapy appears to show higher objective response rates than that in noncisplatin-based therapy, although it is not clear whether it confers a survival benefit. Cisplatin plus doxorubicin had 18 and 49% objective responses, respectively [35, 36]. The combination therapy of cisplatin and mitoxantrone with continuous infusion of 5-FU showed 24 and 27% responses in two different studies [37, 38]. It was reported that 15% of patients showed response rate for cisplatin and epirubicin infusion [39]. Other combination studies reported 24% for cisplatin and doxorubicin plus capecitabine [40] and 6 and 20% in two studies for cisplatin plus capecitabine [41, 42]. The above data suggest that combination chemotherapy may play a minor role and no chemical drug or regimen has been approved for the treatment of HCC [43].

## 3. Molecular-Targeted Therapy

Treatment approaches are now directed against a specific molecular defect, which is termed “molecular-targeted therapies.” Although the molecular pathogenesis of HCC remains poorly understood, the following signaling pathways are notably involved: (1) the epidermal growth factor receptor (EGFR)/EGF (HER1) in the carcinogenesis and proliferative behavior of HCC [44–49]. (2) HCCs are highly vascular tumors with high levels of expression of vascular endothelial growth factor (VEGF). (3) Raf/MAP kinase-ERK kinase (MEK)/extracellular signal-regulated kinase (ERK) pathway is implicated in HCC tumorigenesis [50, 51]. Therefore, small molecule tyrosine kinase inhibitors (TKIs) or monoclonal antibodies (mAbs) to be the target-specific antigens have been proposed and approved by the FDA for clinical use (Table 1). Among them, only sorafenib is approved for treating HCCs. Sorafenib (Nexavar) is a multitargeted orally active TKI, inhibiting Raf kinase and the VEGFR intracellular kinase pathway [52].

## 4. Oncolytic Virotherapy for HCC Treatment

Oncolytic viruses (OVs) have been recently recognized as an effective treatment for cancer in preclinical models and promising clinical responses in human cancer patients [53]. OVs have a number of advantages over conventional antitumor agent, because they have their own cancer specificity and better safety margin. They selectively target and replicate in cancer cells, as a host cell; thus, OVs survive by lysing cancer cells [54]. OV-mediated oncolysis not only leads to tumor regression but also provides important immune responses. Key signals provided by oncolysis to dendritic cells (DCs) and other antigen-presenting cells (APCs) can then initiate additional potent antitumor immune response [55]. In addition to its oncolytic characteristics, OV can be engineered to express some functional genes. For instance, granulocyte macrophage colony-stimulating factor (GM-CSF) expression in OVs increase tumor cell lysis. GM-CSF is an immune modulator, acts as a paracrine manner on various cells, and recruits circulating neutrophils, monocytes, and lymphocytes to enhance their functions in host defense [56]. As for HCC treatment in clinical trials, adenovirus and vaccinia virus (VV) are mostly used [57, 58].

## 5. Oncolytic Virus-Based Preclinical Studies

Most of the preclinical studies use adenoviruses and vesicular stomatitis virus (VSV) as the vector, due to their natural ability to kill or target HCC cells. For instance, adenoviruses have excellent tropism towards hepatocytes [59], in case of VSV, which has intrinsic ability to infect cancer cells [60]. However, various studies have used other viruses like herpes simplex virus (HSV), measles vaccine virus (MeV), newcastle disease virus (NDV), vesicular stomatitis virus (VSV), and vaccinia virus (VV) [61]. In order to target and enhance therapeutic efficacy of viruses, various genes are engineered in their genome. The anticancer and immunotherapeutic efficacy of these viruses are evaluated in HCC cell lines and animal models (Table 2).

### 5.1. Adenoviruses

Adenoviruses are nonenveloped viruses, consist of double-stranded DNA (dsDNA) about 36 kb in size. There are seven subgroups reported, based on cross-reactivity patterns of neutralizing antibodies, from A to G. These are further classified as serotypes. As of now, 57 serotypes have been reported. The pathogenicity and tissue tropism are similar within the subgroups [62]. Adenovirus 5 (Ad5) subtype has been extensively used in oncolytic virotherapy as a vector [63]. Most of the preclinical studies have used adenovirus as a vector to transfer a transgene. Modifications in *E1A* and *E1B* genes of adenovirus have made it as oncolytic vector against the cancer cells [64]. The *E1A* gene is responsible for inactivation of several proteins, including retinoblastoma, allowing entry into S-phase [65]. The E1B inactivates p53, thus preventing apoptosis. A high resistance to tumor necrosis factor–related apoptosis–inducing ligand (TRAIL) has been reported in the HCC cells [66] whereas TRAIL is known to selectively induce apoptosis in malignant cells.

In order to overcome to this hurdle, adenovirus vector, ZD55, was constructed with -Smac (second mitochondria-derived activator of caspases)/TRAIL as ZD55-Smac/ZD55-TRAIL. The combined antitumor effect of these vectors was evaluated in mice xenograft models and cell lines. A significant regression of tumor size was reported in contrast to partial effect of single treatment of ZD55-Smac or ZD55-TRAIL. Moreover, in vitro-based analysis showed activation of caspases and significant reduction of X-linked inhibitor of apoptosis protein (XIAP) expression [67]. Various transgenes have been inserted in those regions for selective targeting of HCC cells. A dual-regulated adenovirus variant CNHK500, in which human telomerase reverse transcriptase (hTERT) drove the Ad5 E1a gene and hypoxia-response promoter controlled the *E1b* gene, was engineered. The efficacy of this virus was checked in HCC cell lines and animal models in terms of tumor targeting, which showed selective gene expression, tumor regression, and prolonged survival period. The results compared with those of CNHK300 confirmed antitumor efficacy and selective replication of CNHK500 in HCC models [68].

Another viral strain, a monoregulated adenovirus CNHK300, was constructed with hTERT, and its antitumor efficacy was evaluated in different cell lines. The detection of hTERT expression and cytotoxicity, especially at low multiplicity of infection (MOI), confirmed the oncolytic activity of CNHK300 [69]. ZD55-IFN-*β* was generated by homologous recombination of ZD55 vector with interferon-*β* (IFN-*β*) containing vector. The antitumor efficacy and IFN-*β* expression of ZD55-IFN-*β* were evaluated in HCC cell lines and mice xenograft model. In contrast to Ad5-IFN-*β*-treated model, 100% cytopathic effect was examined in cell lines and more IFN-*β* expression were observed in both ZD55-IFN-*β* received mice and cell lines. This study confirmed the ability of adenovirus as a carrier of potential anticancer genes [70].

SG7011^let7T^ was constructed as adenovirus with microRNA (miRNA), let-7. It was generated as introducing eight copies of let-7 target sites (let7T) into the 39 untranslated regions of *E1A*. A higher selective replication and cytotoxicity were reported in HCC cell lines when compared to normal liver cell lines. These were also verified in animal models [71]. Telomelysin, an adenovirus with hTERT insertion, had selective replication in HCC cell lines. At a low MOI, ranging 0.77–6.35 pfu, Telomelysin caused HCC cell lysis, but not normal liver cells. These results were confirmed in both in vitro in cell culture and in vivo using an immunocompetent in situ orthotopic HCC model [72].

Adenoviruses armed with apoptosis-induced and immune-stimulatory molecules, such as human TRAIL and IL-12, were constructed as Ad-ΔB/TRAIL and Ad-ΔB/IL-12, respectively. The combined antitumor effects of these vectors were evaluated in Hep3B and HuH7 HCC cell lines. In addition, in vivo efficacy was examined in transplanted Hep3B-orthotopic model. A significant reduction in tumor size of orthotopic model and increased HCC cell death in cell culture were reported. Interestingly, this study documented a remarkable suppression of VEGF with infiltration of natural killer cells (NK cells) and antigen-presenting cells (APCs) at tumor microenvironment (TME). Moreover, enhanced apoptosis and upregulation of interferon-*γ* (IFN-*γ*) production were also reported in this study [73]. In addition, a combination of doxorubicin with *α*-fetoprotein- (AFP-) E1A-IRES-E1B bicistronic cassette derived from Ad5, CV890 showed a synergic antitumor effect in vitro as well as in vivo [74].

### 5.2. Herpes Simplex Viruses

Herpes simplex viruses (HSV) consist of dsDNA (154 kb) as genetic material. Cell receptors, such as herpesvirus entry mediator (HVEM), nectin 1, and nectin 2, are used for cell entry. Using HSV as an oncolytic agent, only very few studies were conducted in HCC model. In order to enhance HCC cell-specific tropism, liver-cancer-specific oncolytic virus (LCSOV) was created. It was constructed by linking the essential viral glycoprotein H gene with the liver specific apolipoprotein E (apoE)-AAT promoter. Then, miR-122a complimentary sequence was further inserted to the 3′ untranslated region (3′UTR). In addition, let-7 was also engineered into the same 3′UTR to increase the safety of this virus. The highly selective replication and tumor cell lysis were reported in xenograft as well as in cell culture models [75]. HSV, G47Δ vector was tested for its antitumor efficacy and cytotoxicity in various HCC cell lines. More than 95% cell toxicity was documented in HepG2, Hep3B, and SMMC-7721 cell lines on day 5 with MOI of 0.01. Intratumoral (IT) injection of G47Δ caused reduction in tumor size and prolonged survival of mice [76].

### 5.3. Vaccinia Virus

**Figure 1:**
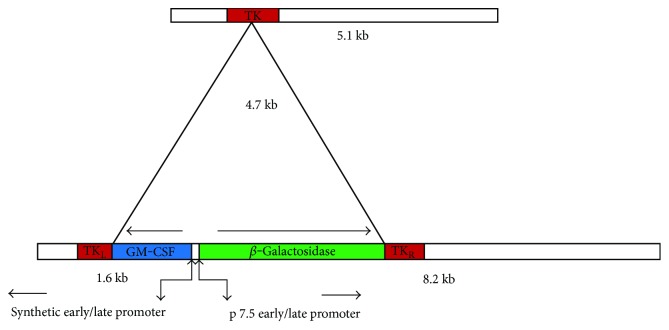
JX-594 was generated as insertion of human GM-CSF and *β*-galactosidase at in between early/late promoter of TK gene. GM-CSF is an immune-stimulatory cytokine, which induces immune response against tumor cells.

VV possesses 190 kb dsDNA as genetic material and widely used in oncolytic virotherapy because of its widest tropism in both mice and human cells [77, 78]. The antitumor and immunotherapeutic efficacies of Wyeth and Lister strains of VV have been demonstrated in preclinical studies. JX-594 from Wyeth strain was constructed by adding human granulocyte microphage colony-stimulating factor (hGM-CSF) and deleting viral thymidine kinase (v*TK*) (Figure 1). Intratumoral (IT)/intravenous (IV) administration of JX-594 in liver cancer models of rabbits and mice showed its anticancer ability against tumors. In addition, eradication of lung metastases from liver tumors was reported in rabbits [79]. The anticancer efficacy of JX-963 (a Western Reserve strain encoding hGM-CSF) was evaluated in HCC, in which rabbits were used as animal models. No significant toxicity was reported in this study. Moreover, a significant increased populations of neutrophils, monocytes, and basophils were found in peripheral blood. In addition, hGM-CSF expression and cytotoxic T cell population were observed at tumor site [80, 81]. The Lister strain, GLV 1h 68, was evaluated for colonization and replication efficiency in HCC. In a xenograft model, a reduction in tumor size and upregulated proinflammatory cytokines level were observed. These results confirmed the antitumor efficacy of GLV 1h 68 against HCC [82]. Interestingly, its efficacy was not affected in sorafenib-resistant HCC cell lines. Antitumor effect of GLV 1h 68 against sorafenib-resistant HCC cells was also observed in vitro. The replication efficacy and infectivity of virus showed similar results in sorafenib-resistant HCC cells when compared to parental HCC cells [83]. GLV-2b372 was generated by inserting *TurboFP635* into *TK* locus of LIVP 1.1.1 cassette for real time monitoring of viral infection. Infectivity, selective replication, biodistribution, and antitumor efficacy were evaluated in various cell panels. 80% of cell death was seen in dose- and concentration-dependent manner in cell lines. Significant viral presence was detected at tumor sites. IT injection of viral strain reduced tumor size in athymic nude mice as a flank xenograft model [84].

### 5.4. Vesicular Stomatitis Virus

VSV consists of single strand RNA (ssRNA) as a genetic material. Due to its intrinsic infectivity against cancer cells, most of the earlier preclinical studies used VSV for oncolytic virotherapy. VSV was recombined with green fluorescence protein (GFP) to examine antitumor efficacy and toxicity in HCC cell panels. The efficient selective replication and cytotoxicity were documented. IT injection of the virus in rat liver showed tumor destruction and tumor growth inhibition, which leads to prolonged survival of animals [85]. Hepatic arterial infusion (HAI) of rVSV-*β*-gal into Buffalo rats bearing orthotopically implanted multifocal HCC showed efficient viral transduction, tumor-selective viral replication, and extensive oncolysis. There were no significant vector-associated toxicities and damage to the hepatic parenchyma reported, whereas prolonged survival was reported in vector-treated group [86]. Recombinant VSV was generated by insertion of a transcription unit expressing a control or fusion protein derived from NDV. Extensive syncytia formation and enhanced cytotoxic effects were observed in both in vivo and in vitro models. Interestingly, no toxicities were found in liver and parenchymal tissues [87]. Antiviral actions of alpha/beta interferon (IFN-*α*/*β*) was checked in rVSV-NDV/F- (L289A-) treated HCC cell lines and rat models. Potential replication efficacy and no toxicity were documented as major outcomes. This study reported an increased index of oncolytic VSV virotherapy in interferon-treated advanced HCC models [88]. In order to improve the oncolytic potency of VSV, VSV (MΔ51) was modified as rVSV (MΔ51)-M3, vector expressing M3, a chemokine-binding protein with broad-spectrum and high affinity from murine gammaherpesvirus-68. This vector was used to treat rats bearing multifocal lesions of HCC. Treatment resulted in a significant reduction of neutrophil and natural killer cell accumulation in the lesions, a 2-log elevation of intratumoral viral titer with enhanced necrosis. There were no apparent systemic and organ toxicities reported in the treated animals [69].

### 5.5. Other Oncolytic Viruses

The effect of the combination of an oncolytic MeV with the novel oral HDACi resminostat (Res) was checked in HCC cell panels. The combination effect showed a boosted cytotoxic effect as an enhanced induction of apoptosis with improved rate of primary infections [89]. In order to enhance therapeutic efficacy, a novel NDV vector harboring an L289A mutation within the F (fusion protein) gene was generated; membrane fusion and cytotoxicity were examined in HCC cell lines and orthotopic liver tumors. Tumor-specific syncytia formation and necrosis without toxicity to neighboring parenchyma were observed. Furthermore, when compared with control NDV, the improved oncolysis conferred by the L289A mutation was translated to significantly prolonged survival of rNDV/F- (L289A-) treated mice [90].

## 6. Oncolytic Virus and Immunotherapy-Based Clinical Trials for HCC

Despite a number of OVs were tried in the preclinical studies, only a few among them have entered into the clinical studies (Table 3). The reasons are different ranging from biodistribution to toxicity. Adenovirus-based trials showed failure in reduced disease progression, but good tolerability of *dI*1520 vector [91]. Most of the studies used vaccinia such as JX-594 from Wyeth strain for the treatment of HCC patients due to its remarkable outcomes in preclinical and clinical trials [77, 78, 81, 92]. The phase I studies have confirmed the anticancer efficacy and immunotherapeutic effect of JX-594 in HCC. The safety and maximum tolerated dose (MTD) were evaluated in liver cancer patients. IT administration in metastatic primary liver tumors showed a significant regression in tumor size. The replication of JX-594 and GM-CSF expression were also confirmed in this study. As the adverse events, grade I–III flu-like symptoms grade, dose-related thrombocytopenia, and grade III hyperbilirubinaemia were experienced by patients when received 1 × 10^9^ pfu [93]. In order to demonstrate antivascular and immunostimulatory properties of JX-594, IT administration in three hepatitis B virus- (HBV-) infected HCC patients was done. This study showed some interesting outcomes, along with the induction of antivascular cytokines and distant tumor targeting, with it suppressed HBV infection [93].

A sequential therapy of JX-594, followed by sorafenib, a multikinase inhibitor and antagonist to vascular endothelial growth factor receptor (VEGFR) in three HCC patients, showed well-tolerance, associated with significantly decreased tumor perfusion and tumor responses (Choi criteria; up to 100% necrosis) [92]. In addition, phase II randomized dose-finding clinical trials showed that survival duration of patients was significantly related to viral dose. The results were confirmed by objective intrahepatic modified response evaluation criteria in solid tumors (mRECIST) (15%) and Choi (62%) response rates. Furthermore, intrahepatic disease control (50%) were equivalent in injected site and distant noninjected tumor sites at both doses. Infusion of low- or high-dose JX-594 into liver tumors (days 1, 15, and 29) were used in this study [81].

## 7. Summary and Future Perspectives

**Figure 2:**
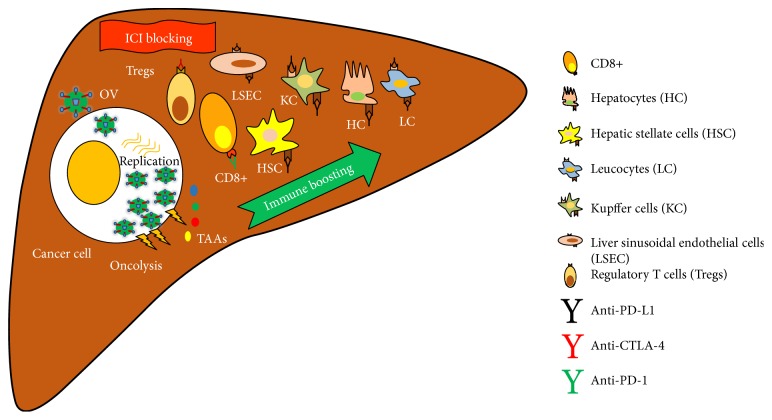
Future prospective for HCC treatment: combination therapy of oncolytic virus with immune checkpoint inhibitor (ICI) blockades. Oncolytic virus enters into cancer cells and replicates in cytosol. It causes oncolysis and activation of neoantigens, which was released from lysed cancer cells. This phenomenon activates immune mechanism against surrounding cancer cells. ICI blockades inhibits action of CTLA-4, PD-L1, and PD-L2 in the liver cells.

Due to limitations in conventional therapies for HCC, new treatment approaches have been established using modern molecular techniques. Oncolytic virotherapy is using oncolytic viruses which selectively infect and kill cancer cells. In order to enhance immunity against cancer cells, genes elevating onco-immunity have been engineered within oncolytic virus. Past and ongoing clinical trials have investigated anticancer efficacy and toxicities of oncolytic viruses, particularly JX-594 (GM-CSF encoding VV) for HCC treatment. GM-CSF as an immune-stimulatory cytokine boosts host immune activity through the infiltration of dendritic cells (DCs) and CD4+ and CD8+ T cells at tumor sites. Despite these outcomes have shown favorable results for prognosis of HCC treatment, as an immunotherapeutic approach, more advanced methods, that is, combination therapy with immune checkpoint inhibitors (ICIs) such as monoclonal antibodies (mAbs) against cytotoxic T-lymphocyte-associated antigen 4 (CTLA-4), programmed death-ligand 1 (PD-L1), and/or programmed cell death protein 1(PD-1), would be taken into the account (Figure 2). This is because of “tolerogenic” nature of the liver; it expresses more immune checkpoints associated molecules. At present, antitumor efficacy of immune checkpoint inhibitors (ICIs) drugs like Nivolumab and Ipilimumab are being evaluated in various clinical trials. Completed trial results showed positive outcomes in HCC treatment [94]. Therefore, combination therapy approaches as oncolytic virotherapy with immune checkpoint blockades may accelerate treatment. Moreover, genetically engineered oncolytic virus with mAbs can be generated for advancement treatment in HCC models. Development of biomarker to monitor the treatment outcomes and toxicities would be very helpful in the translational medicinal research.

## Figures and Tables

**Table 1:** TKI and mAbs approved by FDA for use of cancer therapy.

Category	Name	Targets	Uses
TKI	Dasatinib	BCR-ABL, SRC family, c-KIT, PDGFR	Chronic myeloid leukemia (CML), acute lymphocytic leukemia
	Erlotinib	EGFR	Non-small-cell lung cancer (NSCLC), pancreatic cancer
	Gefitinib	EGFR	NSCLC
	Imatinib	BCR-ABL, c-KIT, PDGFR	Acute lymphocytic leukemia, CML, gastrointestinal stromal tumor
	Lapatinib	HER2/neu, EGFR	Breast cancer
	Sorafenib	BRAF, VEGFR, EGFR, PDGFR	Renal cell carcinoma (RCC), hepatocellular carcinoma (HCC)
	Sunitinib	VEGFR, PDGFR, c-KIT, FLT3	RCC, gastrointestinal stromal tumor
	Temsirolimus	mTOR, VEGF	RCC
	Pazopanib	VEGFR-1, VEGFR-2, VEGFR-3, PDGF-*α*/*β*, and c-KIT	RCC
	Nilotinib	BCR-ABL	CML
	Crizotinib	ALK, HGFR	NSCLC
	Vemurafenib	BRAF	Late-stage melanoma
mAb	Alemtuzumab	CD52	Chronic lymphocytic leukemia
	Bevacizumab	VEGF	Colorectal cancer, NSCLC, RCC
	Cetuximab	EFGR	Colorectal cancer, head and neck cancer
	Gemtuzumab ozogamicin	CD33	Relapsed acute myeloid leukemia
	Ibritumomab tiuxetan	CD20	Non-Hodgkin's lymphoma (NHL) (with yttrium-90 or indium-111)
	Panitumumab	EGFR	Colorectal cancer
	Rituximab	CD20	NHL
	Tositumomab	CD20	NHL (with iodine-131)
	Trastuzumab	HER2/neu	Breast cancer with HER2/neu overexpression
	Ipilimumab	CTLA-4	Late-stage melanoma

**Table 2:** Representative OVs used in preclinical studies.

Virus strain	Modification	Therapeutic gene	HCC cell lines used	Animal model	Dose (pfu) and route	Reference
Adenovirus, CV890	*AFP* transcriptional regulatory elements (TRE) to control an artificial E1A-IRES-E1B bicistronic cassette in an ad5 vector	None, combination with doxorubicin	HepG2, Huh7, Hep3B, and SNU449	BALB/c nude mice Hep3B/HepG2 xenografts	1 × 10^11^ IV	[74]
Adenovirus, ZD55-TRAIL/ZD55- Smac	Deletion of *E1B* 55KDa gene Insertion of *EGFP, TRAIL*/*Smac* genes	*TRAIL*/*Smac*	Hep3B, BEL7404, and SMMC7721	BALB/c nude mice BEL7404 xenograft	2 × 10^9^ IT	[67]
Adenovirus, CNHK500	Human telomerase reverse transcriptase (*hTERT*) and hypoxia-response promoter controlled the *E1b* gene	*hTERT*	Hep3B, HepGII, and SMMC-7721	BALB/c nude mice, SMMC-7721, and Hep3B xenografts	2 × 10^8^–2 × 10^9^ IT	[68]
Adenovirus, CNHK300	*hTERT* was inserted with viral genome at upstream of *E1A* region	*hTERT*	HepGII and Hep3B	No animal model used	MOI of 5 pfu/cell	[69]
Adenovirus, ZD55-IFN-*β*	E1B*-*55-kDa gene deletions and *hIFN*-*β* insertion	*hIFN*-*β*	Hep-G2 and BEL7404	BALB/c nude mice, BEL7404 xenografts	2 × 10^9^ IT	[70]
Adenovirus, SG7011^let7T^	Insertion of eight copies of let-7 target sites (let7T) into the 39 untranslated region of *E1A*	miRNA, *let*-*7*	HepG2, Hep3B, PLC/PRF/5, and Huh7	BALB/c nude mice, Hep3B and SMMC-7721 xenografts	5 × 10^8^ IT	[71]
Adenovirus, Telomelysin	*hTERT* inserted upstream of the *E1* gene	*hTERT*	Human: Huh-7, Hep3B, PLC5, HA22T, HCC36, and HepG2 Mouse: Hepa-1c1c7 and Hepa 1–6	HBx transgenic mice, orthotopic model	Low: 1.25 × 10^8^ Medium: 6.25 × 10^8^ High: 3.0 × 10^9^ IT	[72]
Adenovirus, Ad-ΔB/TRAIL and Ad-ΔB/IL-12	Mutated in *E1A* and deleted in *E1B* regions. Insertion of *hTRAIL* or *hIL*-*12*	*hTRAIL* or *hIL*-*12*	Hep3B and HuH7	Athymic nude mice, orthotopic model	2 × 10^8^ 1 × 10^10^ IV	[73]
HSV, designated liver-cancer specific oncolytic virus (LCSOV)	Viral glycoprotein H gene linked with liver-specific apolipoprotein E (apoE)-AAT promoter. miR-122a complimentary sequence to the 3′ untranslated region (3′UTR). miR-124a and let-7 also inserted at 3′ UTR	*miR122miR*-*124a* and *let*-*7*	HuH-7, HepG2, and Hep3B	Hsd: athymic (nu/nu) mice, Hep3B xenograft	5 × 10^6^ IT	[75]
HSV, G47Δ	ICP47 and *γ*34.5-deletion	None	HepG2, HepB, SMMC-7721, BEL-7404, and BEL-7405	Balb/c nude mice SMMC-7721, BEL-7404 xenograft	2 × 10^7^ IT	[76]
MeV, (Res + MeV)	Encoding of *GFP* as a marker gene and *SCD* as suicide gene	None Combination with HDACi drug resminostat (Res)	HepG2 and Hep3B	No animal model used	Various MOIs	[89]
NDV	L289A mutation within the *F* gene	None	HepG2 and Huh7	Buffalo rats McA-RH7777 rat HCC-orthotopic model	10^8^ TCID_50_/rat HAI	[90]
VV, JX-594	Deletion of *TK* and *VGF*, insertion of h *GM*-*CSF*	*hGM*-*CSF*	None	Immunocompetent, orthotopic, NZW rabbits. VX2 tumor model. Rat: chemically induced HCC	Rabbit: 1 × 10^8^–1 × 10^9^ IV/IT Rat: 1 × 10^8^ IT	[79]
VV, JX-963	Deletion of *TK* and *VGF*, insertion of *h GM*-*CSF*	*hGM*-*CSF*	None	Immunocompetent, orthotopic, NZW rabbits VX2 tumor model	Various IV	[80]
VV, GLV-1 h68	Deletion of *TK* and insertion of Renilla luciferasegreen fluorescent protein (*Ruc*-*GFP*), *β*-*galactosidase*, *β*-*glucuronidase*	None	HuH7 and PLC/PRF/5	Athymic Nude-*Foxn1*^nu^ HuH7 and PLC xenografts	5 × 10^6^ IV	[82]
VV, GLV-1 h68	Deletion of TK and insertion of Renilla luciferasegreen fluorescent protein (Ruc-GFP), *β*-galactosidase, *β*-glucuronidase	None	Huh-7, Hep 3B, SNU-449 and SNU-739	No animal model used	MOI of 0.001, 0.01, 0.1, and 1	[83]
VV, GLV-2b-372	Deletion of *TK* and insertion of *TurboFP635* gene	None	Huh-7, Hep G2, SNU-449, and SNU-739	Athymic nude mice Huh-7 xenograft	1 × 10^5^ IT	[84]
VSV, rVSV-GFP	Insertion of *GFP*	None	Human: Hep 3B and Hep G2 Rat: McA-RH7777	Buffalo rats McA-RH7777 orthotopic, syngeneic	1 × 10^8^ IT	[85]
VSV, rVSV-*β*-gal	Insertion of *β*-galactosidase (*β*-*gal*)	None	McA-RH7777	Buffalo rats McA-RH7777 orthotopic	1.3 × 10^7^ HAI	[86]
VSV, rVSV-NDV/F(L289A)	Insertion of *NDV*/*F*	None	Human: Hep 3B and Hep G2 Rat: McA-RH7777	Buffalo rats, orthotopic syngeneic McA-RH7777	1.3 × 10^7^ HAI	[85–87]
VSV, rVSV-NDV/F(L289A)	Insertion of *NDV*/*F*	None	Human: Hep 3B and Hep G2 Rat: McA-RH7777	Buffalo rats, orthotopic syngeneic McA-RH7777	1.3 × 107 HAI	[88]
VSV, rVSV(MΔ51)-M3	MΔ51deletion and M3 addition	None	McA-RH7777	Buffalo rats McA-RH7777 orthotopic	5.0 × 10^7^–5.0 × 10^9^ HAI	[69]

IV: intravenous; IT: intratumoral; MOI: multiplicity of infection.

**Table 3:** Clinical trial outcomes in HCC patients.

Virus strain	Modification	Phase	Dose (pfu)	Route	Outcomes	Reference
Adenovirus, *dl*1520	*E1B* deletion	I	3 × 10^11^	IV	Tolerability was shown in patients but failed to reduce the disease progression	[91]
Vaccinia virus, JX-594	Deletion of *TK* and *VGF*, insertion of human *GM*-*CSF*	I	10^8^, 3 × 10^8^, 10^9^, or 3 × 10^9^	IT	Well toleration, 1 × 10^9^ pfu, was maximum-tolerated dose (MTD) and hyperbilirubinemia noted as the dose-limiting toxicity	[93]
Vaccinia virus, JX-594	Deletion of *TK* and *VGF*, insertion of human *GM*-*CSF*	I	3 × 10^6^	IT	Induction of antivascular cytokines and suppressed HBV replication in the patients	[93]
Vaccinia virus, JX-594	Deletion of *TK* and VGF, insertion of human *GM*-*CSF*	I	10^8^ or 10^9^	IT	Safety and efficacy of JX-594 followed by sorafenib: well toleration and tumor perfusion	[92]
Vaccinia virus, JX-594	Deletion of *TK* and VGF, insertion of human *GM-CSF*	II	10^8^ or 10^9^	Intravascular fusion	Subject survival duration was significantly related to high dose of JX-594	[81]
